# Agrochemical exposure-induced seed microbiome response in barley

**DOI:** 10.1007/s44297-023-00013-w

**Published:** 2023-11-29

**Authors:** Lan Wang, Hongda Fang, Zhao Xue, Ji De, Xiaofang Guo

**Affiliations:** 1 School of Environmental Ecology, Tibet University, Lhasa, 850000 China; 2 State Key Laboratory of Rice Biology and Breeding, Zhejiang University, Hangzhou, 310058 China

**Keywords:** Seed microbiota, Secific response, Agrochemicals, Ecological risk, Biomarkers, Highland barley

## Abstract

The seed microbiota has great potential for enhancing agricultural practices and promoting sustainable plant growth. However, excessive and improper application of agrochemicals may have unforeseen effects on the seed microbiota, posing potential risks to crop health and even ecological integrity. Despite this, the impact of agrochemicals on seed microbiota in domesticated crops remains largely unexplored. Here, we aimed to analyze the effects of four common agrochemicals on the seed microbiome of highland barley in Tibet using high-throughput sequencing. Community diversity analysis suggests that the differences in seed microbial groups under different agrochemical treatments are not significant. Interestingly, we found that the key microbial taxa in barley seeds responded distinctly to specific agrochemicals. *Lactobacillus* spp. were identified as positive biomarkers in response to tebuconazole and lambda-cyhalothrin due to an increase in relative abundance as a probiotic. Conversely, *Leuconostoc* and *Helicobacter* were recognized as positive biomarkers upon exposure to pydiflumetofen, attributed to a notable reduction in the abundance of pathogens. Collectively, our findings have revealed the unique responsiveness of seed microbial taxa to agrochemical exposure. Further mechanistic insights into the responsiveness of these microbial biomarkers to agrochemicals may establish a foundational framework for microbiome-targeted verification of agrochemical application, ensuring food safety throughout the cultivation of Tibetan barley.

**Supplementary Information:**

The online version contains supplementary material available at 10.1007/s44297-023-00013-w.

## Introduction

The plant microbiome refers to the diverse community of microorganisms that reside within and surrounding plant tissues [1]. It plays a crucial role in regulating plant health and adaptability [2, 3]. Over the years, extensive research has revealed the significant impact of the plant microbiome on various aspects of plant life, such as nutrient uptake, disease resistance, and stress tolerance [4–7]. Among the intriguing components of the plant microbiome is the seed microbiome, which has gained substantial attention in recent years [8, 9]. The seed microbiome consists of microorganisms associated with the seed surface or internal tissues, and it has been found to influence seed germination, seedling vigor, and early plant development [10]. As previous studies reported, the potential of five endophytic bacteria isolated from wheat seeds to enhance wheat’s disease resistance has been highlighted, while research on maize seeds has unveiled a correlation between the composition of the endophytic bacterial community and the host plant’s phylogeny [11–13]. Harnessing the functional traits of the plant microbiome, particularly the seed microbiome, holds great promise for enhancing agricultural practices and fostering sustainable plant growth strategies [14–16].

Since the Green Revolution in the 1960s, global agrochemical inputs have steadily increased and are currently considered the most reliable solution to ensure food supply for a growing population [17]. In recent decades, the usage of agrochemicals in agriculture has shown a significant increasing trend, primarily driven by the rise in pest and disease epidemics caused by climate change [18, 19]. While these artificial chemicals have contributed to higher crop yields and effective pest and disease control, their excessive and improper use has raised concerns about ecological risks [20–22]. This has led to the development of resistance in pests and pathogens and negative impacts on the non-target organisms, such as beneficial insects, birds, and aquatic life [23–25]. The seed microbiota consists of microorganisms associated with the seed surface or internal tissues, which have been reported to affect seed germination, seedling vigor, and early plant development [26–28]. The extensive application of agrochemicals may exert unforeseen effects on plant health and even ecological integrity, although it remains largely unexplored in domesticated crops [17, 22, 29].

Highland barley (*Hordeum vulgare var. nudum (L.) Hook.f., qingke*) is considered the earliest crop domesticated by humans in Tibet, China. It has thrived and become a dominant crop, particularly at high altitudes ranging from 4200 to 4500 m [30]. Currently, it has garnered significant attention as a natural and wholesome grain due to its unique nutritional components around the world, leading to significant economic value and potential for market development [31]. The seed microbiome, which is highly conserved in different types of qingke seeds, may promote host growth or assist the host in resisting pests and diseases [30, 32]. In this study, we employed 16S rDNA-based high-throughput sequencing to profile the seed microbiome in barley seeds after exposure to four representative agrochemicals. These pesticides were primarily used in cultivated regions and exemplify diverse mechanisms of action and distinct chemical structures. Specifically, imidacloprid and lambda-cyhalothrin serve as insecticides, whereas pydiflumetofen and tebuconazole function as fungicides. This research provides a valuable basis for understanding the impact of these common agrochemicals on the seed microbiome in domesticated crops.

## Results

### Diversity of the seed-associated bacterial community under different pesticide exposures

**Fig. 1:**
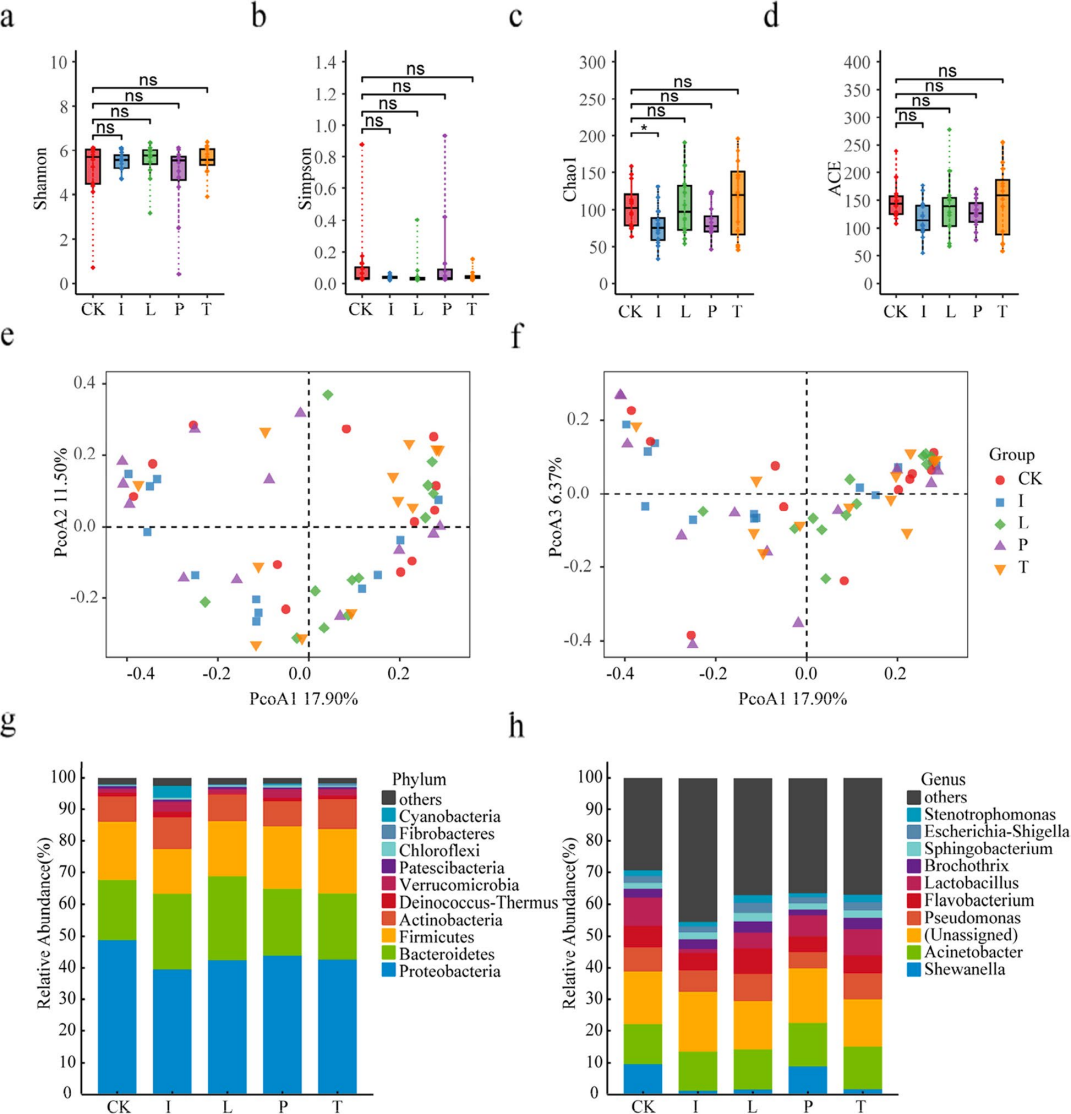
Analysis of the seed microbiome in Qingke treated by representative agrochemicals. **a**-**d** Comparison of bacterial community diversity between the seed bacteria in Qingke seeds under different treatments was implemented by analysis of Shannon (**a**), Simpson (**b**), Chao1 (**c**), and ACE (**d**) indices. Significant difference values are shown by “*” according to the Kruskal–Wallis test and Dunn's test. Data repeatability was tested by using PCoA of the bacterial communities. **e**, **f** Bacterial community structures were analysed in five groups: exposure to blank control, imidacloprid (I), lambda-cyhalothrin (L), pydiflumetofen (P), and tebuconazole (T). The bacterial community was visualized at the phylum (**g**) and genus (**h**) levels

To investigate the structure of resident bacterial communities under representative agrochemicals. Alpha diversity analyses indicated that the evenness of bacterial communities was not significantly altered during the treatment with various agrochemicals, according to the Simpson and Shannon indices (Fig. 1a and b). It was also observed that the species richness was not substantially distinct between the unexposed seeds and others, despite a decrease in richness under imidacloprid exposure (Fig. 1c and d). Moreover, beta diversity-based principal coordinate analysis (PCoA) performed by Bray‒Curtis distance and Permutational Multivariate Analysis of Variance (PERMANOVA) further revealed an unnotable difference between exposed and control seeds in bacterial communities (Fig. 1e and f, Supplementary Table 1). In terms of the bacterial structure at the phylum level, the dominant phyla of the control seeds followed a decreasing order of Proteobacteria (48.7%) > Bacteroidetes (19.0%) > Firmicutes (18.3%) > Actinobacteria (8.2%) (Fig. 1g). When the seeds were exposed to typical agrochemicals, the bacterial structures were found to be of a similar composition as in differently treated seeds despite the changes in the abundance of each phylum (Fig. 1g). Exposure to specific agrochemicals resulted in significant alterations in the dominant bacterial taxa (> 5%) within Qingke seeds at the genus level. For instance, Shewanella, a prominent genus in the control group, lost its dominance in the imidacloprid-, lambda-cyhalothrin-, and tebuconazole exposure groups. Notably, pydiflumetofen exposure caused changes in the dominant taxa, with Flavobacterium losing its dominance (Fig. 1h). These observations suggest that the assembly of the microbial community was characterized by different tendencies in bacterial taxa, which indicated that the responses of the seed microbiome to agrochemicals might be highly specific.

### The key microbial taxa distinctively respond to agrochemical-specific types in barley seeds

**Fig. 2:**
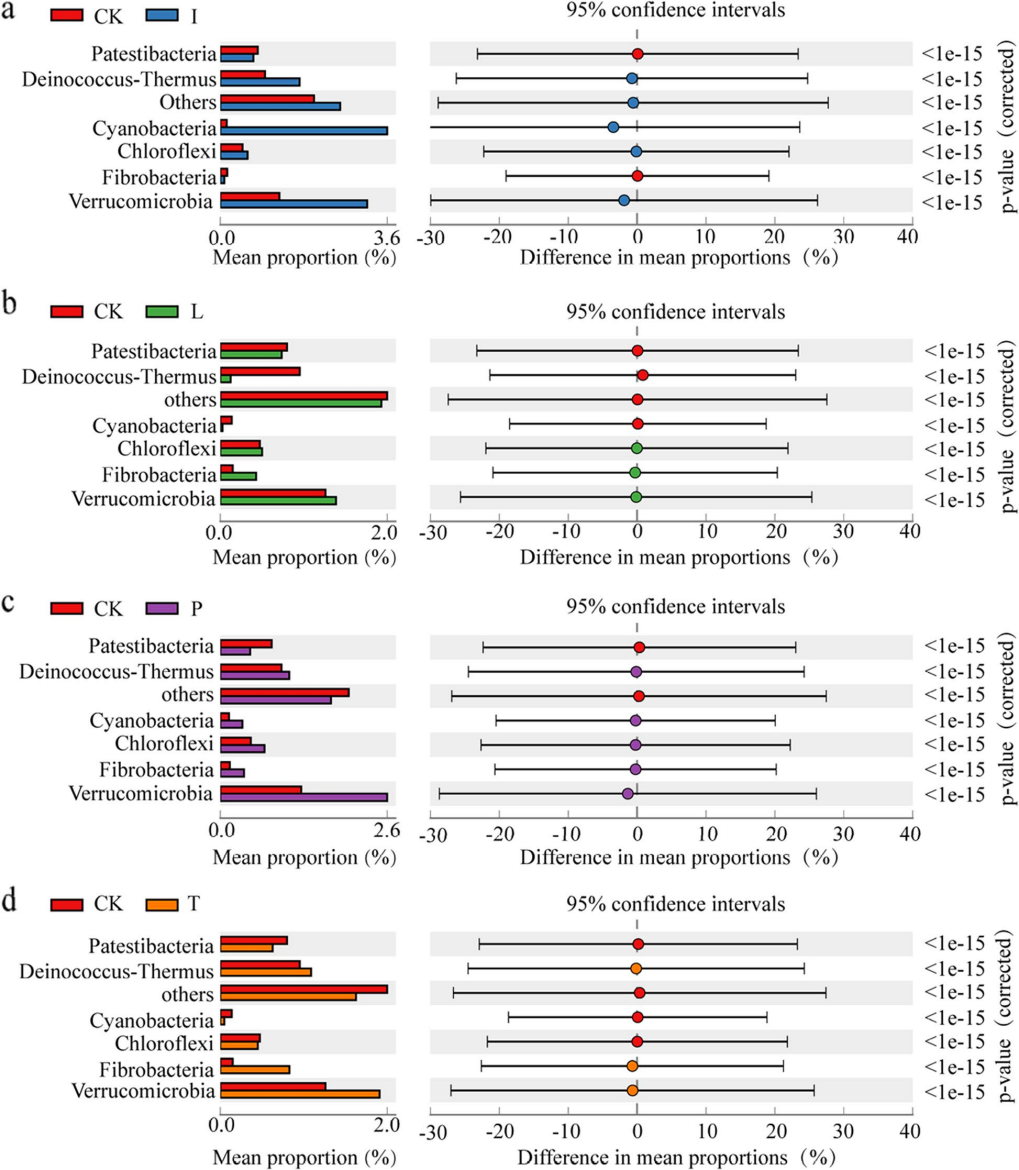
Comparative analyses of barley seed bacterial phyla under different agrochemical exposures. Variations in relative abundance under imidacloprid (**a**), lambda-cyhalothrin (**b**), pydiflumetofen (**c**), and tebuconazole (**d**) exposure

To investigate the key microbial taxa in the microbial community that demonstrate specific responses to representative agrochemicals, we conducted differential analysis based on clustered relative abundances. At the phylum level, significant differences emerged between the control and agrochemical-exposed groups for Patescibacteria, Deinococcus-Thermus, Cyanobacteria, Chloroflexi, Fibrobacteres, and Verrucomicrobia (Fig. 2a and b). Interestingly, we noticed that different phyla had inconsistent responses to different agrochemichals. As an illustration, the abundance of Deinococcus-Thermus significantly decreased solely under lambda-cyhalothrin exposure but showed a notable increase under other treatments (Fig. 2b). When the bacterial community was assessed at the genus level, it was shown that exposure of seeds to imidacloprid resulted in a significant increase in Brochothrix and Sphingobacterium abundance, while specifically decrease in the proportion of Lactobacillus (Supplementary Fig. 1a). Exposure to lambda-cyhalothrin significantly increased the relative abundance of Brochothrix, Escherichia-Shigella, Stenotrophomonas, and Sphingobacterium, while decreasing the relative abundance of Shewanella (Supplementary Fig. 1b). Pydiflumetofen exposure resulted in a decrease in the abundance of the four taxa, and tebuconazole exposure exhibited a similar tendency to that observed with seeds exposed to lambda-cyhalothrin (Supplementary Fig. 1c and d). Moreover, diverse pesticide exposures induced notable fluctuations in the relative abundance of non-dominant phyla, including Cyanobacteria, Chloroflexi, and Fibrobacteres (Figs. 1g and 2). Additionally, less prevalent genera, such as Brochothrix, Escherichia-Shigella, and Stenotrophomonas, displayed significant shifts in relative abundance due to pesticide exposure (Fig. 1h, Supplementary Fig. 1). The differing responses to representative agrochemicals suggest that non-dominant taxa might exhibit heightened sensitivity to non-biological stressors, in comparison to their dominant counterparts.

### Tracking of specific-response biomarkers in the resident microbiome of barley seeds

**Fig. 3:**
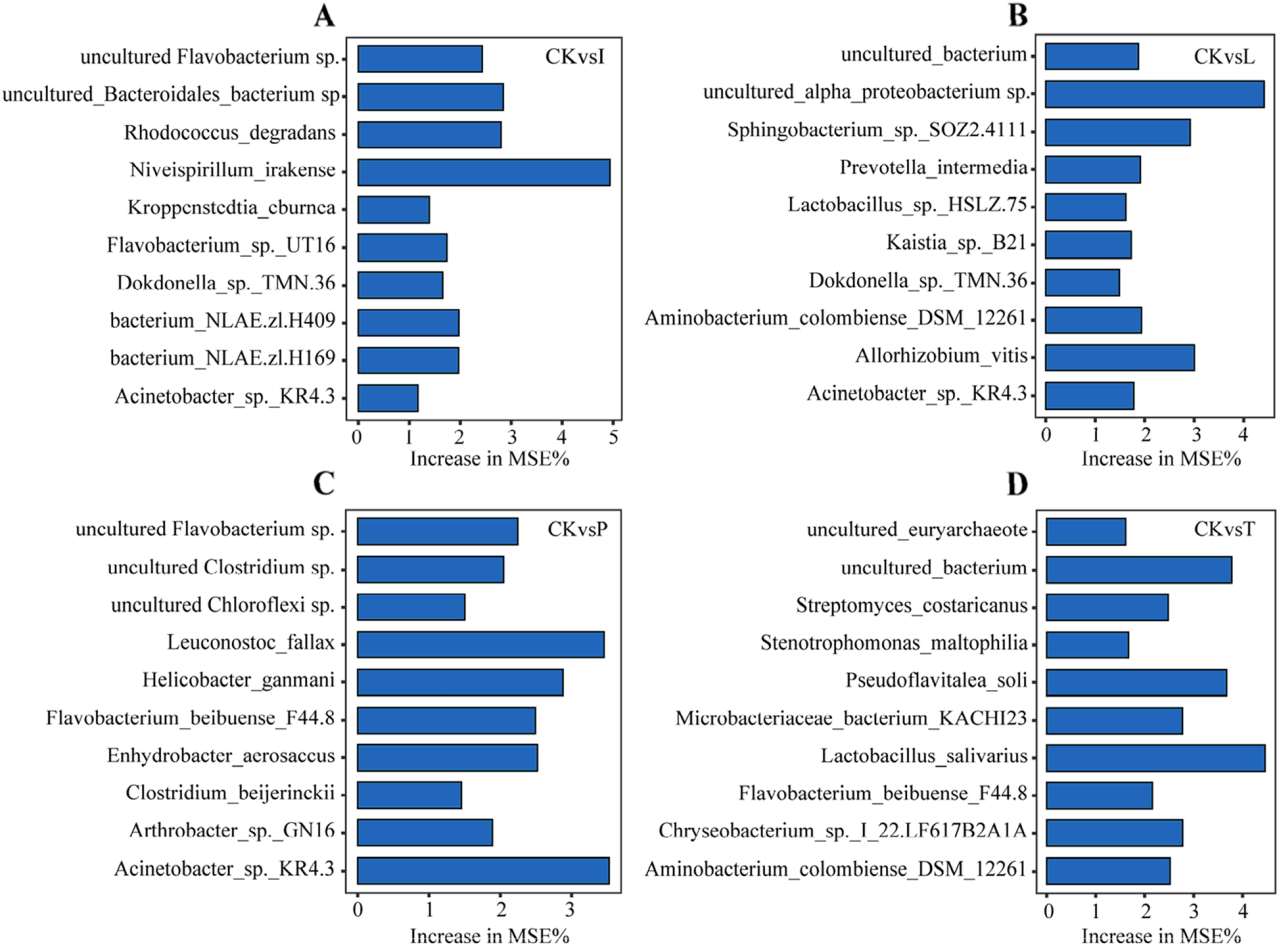
Random forest model detects bacterial taxa that accurately predict biomarkers. **a** Comparison between control and imidacloprid-treated cells. **b** Contrasting lambda-cyhalothrin exposure with the control. **c** Assessment of the control group compared to the pydiflumetofen exposure. **d** Evaluation of differences between the control group and tebuconazole exposure

For further confirmation of the relationship between control and exposure to agrochemicals with microbiome data at the feature levels, we established a model using a random-forest machine-learning method. We deduce that the essential species can be identified as bacterial biomarker candidates, which have been subject to distinct agrochemical impacts (Fig. 3). The imidacloprid exposure group exhibited six potential bacterial biomarkers, including *Niveispirillum irakense* and *Rhodococcus degradans* (Fig. 3a), while the lambda-cyhalothrin group showed eight biomarkers, including *Sphingobacterium* sp. SOZ2.4111 and *Aminobacterium colombiense* DSM 12261 (Fig. 3b). Pydiflumetofen and tebuconazole treatments resulted in seven and eight potential biomarkers, respectively, such as *Acinetobacter sp. KR4.3* and *Lactobacillus salivarius* (Fig. 3c, d). Nevertheless, to further verify whether these indicators were positive or negative, we needed to analyze changes related to relative abundance (Supplementary data 1). More specifically, due to the index among candidates and the undetectable reads when seeds were exposed to agrochemicals, *Niveispirillum irakense* experienced suppression within the community following the use of imidacloprid (Fig. 3a). *Lactobacillus salivarius* and *Lactobacillus* sp. HSLZ-75 was recognized as a positive biomarker in tebuconazole and lambda-cyhalothrin (Fig. 3b and d). Seeds treated with pydiflumetofen, *Leuconostoc fallax* and *Helicobacter ganmani* were acknowledged as positive biomarkers. (Fig. 3c). However, the underlying mechanisms by which biomarkers differ in response to representative agrochemicals of highland barley remain to be elucidated.

## Discussion

The plant microbiome has a significant impact on plant growth to improve plant adaptability to different environments [33]. Seeds are a vital part of plants, and the functional features of the seed microbiome have attracted much interest in recent years [8, 34]. However, the increased reliance on agrochemical inputs has boosted food production but raised ecological concerns due to overuse and improper application, which has led to not only concerns about food safety but also disturbances in microbial communities, particularly in seeds [14, 15, 22].

In the present study, the taxonomic composition of the bacterial communities revealed an intriguing pattern of complex links between agrochemicals and microbial communities. The alpha and beta diversity analyses, as indicated by various indices, suggest that the evenness and richness of the bacterial communities did not have significant alterations when subjected to common agrochemical treatments (Fig. 1a-d). At the overall community level, the effects of the various agrochemicals on bacterial composition were relatively subtle (Figs. 1 and 2). Interestingly, we observed varying trends in dominant taxa within the seed microbiome when subjected to representative agrochemicals. The relative abundances of these microorganisms demonstrated fluctuations, displaying increases or decreases in response to different agrochemicals (Supplementary data 1). The appearance of this phenomenon can be attributed to a combination of factors, including the diverse chemical composition of agrochemicals and distinct modes of action, microbial adaptation, complex ecological interactions, and the influence of environmental conditions on microbial communities [14, 18, 35]. Although prominent changes in community structure might not be induced by typical agrochemicals, these chemicals are likely to have a specific impact on bacteria within the community.

Our findings show that some bacterial taxa displayed greater susceptibility to pesticide exposure in contrast to dominant taxa. As previously reported, non-dominant microbial taxa in tobacco leaf bacteria exhibited greater sensitivity to broad-spectrum insecticide exposure, which could be attributed to the ecological roles and lower population densities of these non-dominant taxa within the environment [36, 37]. Furthermore, numerous studies have pointed out that abundant microorganisms play a crucial role in nutrient cycling and exhibit stronger environmental adaptability [38, 39]. Therefore, the more abundant groups within the endophytic bacteria in seeds show greater "resistance" to agrochemicals. A specific microorganism was very recently found to play significant roles in activating the host’s metabolism defence and in promoting resistance against a globally prevalent phytopathogen to maintain plant ecosystems [7, 40, 41]. The application of tebuconazole and lambda-cyhalothrin led to the enrichment of *Lactobacillus* spp., potentially enhancing plant disease resistance (Fig. 3b and d). *Niveispirillum irakense* plays a physiological role in the ecological environment, which contributes to enhancing plant stress tolerance and immune responses and could be identified as a negative biomarker (Fig. 3a) [42]. The specific enrichment of *Leuconostoc fallax* in response to pydiflumetofen suggests its potential ability to degrade organic compounds and could serve as a negative biological marker to assess the application of agrochemicals (Fig. 3c). Although they play crucial roles in production, their inherent characteristics make them insensitive to the impacts of antibiotics. This insensitivity could potentially create an indirect hazard for microbial ecosystems, plants, and even humans [43–45]. These uniquely responsive microbes could serve as biomarkers, prompting us to delve deeper into the equilibrium between the application of common agrochemicals, microbial communities, and the well-being of plants and even ecological integrity.

## Conclusions and perspectives

Within the framework of detailed assessment of the impact of seed microbial communities by agrochemicals, we can observe that certain sensitive microbes with biological functions exhibit specific responses. Distinct microbial responders provide us with a valuable point of interaction to uncover the relationship between microorganisms and agrochemical application. Microbial biomarkers associated with pesticides offer diverse applications, spanning agricultural soil monitoring, evaluation of water and soil pollution, and quality assurance of agricultural produce. Consequently, there is a need for comprehensive investigations on a broader scale and across extended timeframes to delve into the underlying mechanisms of microbial biomarkers linked to agrochemical exposure in crops. In conclusion, our findings have revealed the unique responsiveness of seed microbial taxa to agrochemical exposure. Further mechanistic insights into the responsiveness of these microbial biomarkers to agrochemicals will establish a foundational framework for microbiome-targeted verification of agrochemical application, ensuring food safety throughout the cultivation of Tibetan barley.

## Materials and methods

### Field experimental design and sample collection

The field experiment was conducted in Qiangga Township, Linzhou County, Lhasa city, Tibet Autonomous Region (29°57'19" N, 91°9'4" E). The soil type and fertilization conditions were consistent among the experimental plots. To ensure representativeness, four primary qingke varieties, Zangqing 2000, Zangqing 320, Himalaya 22, and 13-5171-7, were selected for the field trial. The pesticide treatments consisted of application of 0.03 g/m2 10% imidacloprid wettable powder (B; Sichuan Guoguang Agrochemical Co., Ltd., Chengdu, China), application of 0.0075 ml/m2 of 10% lambda-cyhalothrin aqueous emulsion (G; Shandong Bainong Sida Biological Technology Co., Ltd., Qinzhou, China), application of 0.06 ml/m2 of pydiflumetofen suspension (F; Syngenta China Investment Co., Ltd., Shanghai, China), and application of 0.03 ml/m2 of tebuconazole (W; Shandong Bainong Sida Biological Technology Co, Ltd., Qinzhou, China). Finally, a blank control (CK) treatment was also included. The experimental field had an area of 60 m2, with 20 plots (2 × 1.5 m) and a 0.5 m buffer zone between plots. The test barley was treated with pesticides using the spraying method in July 2022. This method is consistent with the practices used by surrounding farmers in actual agricultural production. Barley spike samples were collected in September 2022, and 60 samples were sent to Megagenomics Co., Ltd. (Guangzhou, China) for high-throughput sequencing.

### DNA extraction

Barley seeds underwent a series of steps to ensure their surface cleanliness: they were washed with phosphate-buffered saline (PBS) to remove loosely attached material and then subjected to ultrasonication in clean PBS for 2 minutes. Subsequently, the seeds were immersed in a 1% sodium hypochlorite solution for 1 minute, followed by two 1-minute immersions in 75% ethanol. This was followed by two 1-minute soakings in sterile water and a final rinse. After surface sterilization, the barley seeds were frozen in liquid nitrogen and ground using a sterile mortar. Genomic DNA was extracted from the samples using the Plant DNA Extraction Mini Kit B (Mabio Co., Guangzhou, China). The purity and concentration of the extracted DNA were assessed using a NanoDrop One spectrophotometer (Thermo Fisher Technology Co., USA).

### PCR Amplification of the 16S rRNA genes

For PCR amplification, the barcoded primers 515F (5' GTGYCAGCMGCCGCGGTAA) and 806R (5' GGACTACHVGGTTWTAA) were used in conjunction with TaKaRa Premix Taq® Version 2.0 (TaKaRa Biotechnology Co., Dalian, China). The PCR reaction volume was 50 μL, consisting of 23 μL of ultrapure water, 25 μL of Premix Taq (2×), 1 μL of each primer (10 μM), and 50 ng of DNA template. The PCR cycling program encompassed an initial denaturation step at 94°C, followed by 30 cycles of denaturation at 94°C for 30 s, annealing at 52°C for 30 s, extension at 72°C for 30 s, and a final extension at 72°C for 10 min. Each sample was replicated three times, and PCR products from the same sample were pooled. Gel electrophoresis using a 1% agarose gel was conducted to assess the length and concentration of the PCR products. The ensuing PCR products were purified using the E.Z.N.A.® Gel Extraction Kit (Omega, USA) according to the manufacturer's instructions to recover the desired DNA fragments. Finally, PE250 sequencing of the PCR products was carried out using an Illumina Nova 6000 platform at Magigene Co. Ltd. (Guangzhou, China).

### Bioinformatics analysis

The removal of barcodes and adapter sequences, as well as sequence trimming, was conducted using FASTP software (version 0.14.1, https://github.com/OpenGene/fastp). The resultant raw tags were further subjected to filtration through the use of usearch-fastq mergepairs software (version V10, http://www.drive5.com/usearch). Employing the UPARSE method, the filtered data underwent clustering. Subsequently, species annotation was accomplished using the DADA2 and Deblur algorithms, which are components of the QIIME2 pipeline (version 2020.11.0), in conjunction with the SILVA database [46]. The annotation was performed with a default confidence threshold of 0.8.

### Statistical analysis

All statistical analyses and data visualization were conducted using R, version 4.1.1. Any other software used was explicitly mentioned in the text. The vegan package in R was utilized for the computation of microbial alpha diversity indices (including Shannon, Simpson, Chao 1, and ACE indices). Differences in alpha diversity indices among seed-associated bacterial communities across various treatments were analyzed using the Kruskal‒Wallis test followed by Dunn's test. Principal Coordinate Analysis (PCoA) based on the Bray‒Curtis distance was employed to assess the impact of different pesticide exposures on the structure of seed-associated bacterial communities. The OTU table was annotated, and the top ten taxa at both the phylum and genus levels, as well as other taxa, were visualized by the ggplot2 package. The relative abundance changes in seed-associated bacteria under different pesticide exposures were analyzed using STAMP software. For the identification of sensitive seed-associated bacterial biomarkers in response to pesticide exposure, the relative abundances of bacterial taxa at the species level of Qingke seed-associated bacteria were classified using Random Forest package in R with default parameters.

## Supplementary Information

**Additional file 1:** **Supplementary Table 1.** PerMANOVA test results for internal seed bacterial community in Qingke seeds under different pesticide exposures. **Supplementary Fig. 1.** Comparative analyses of seed bacteria at genus level in response to various agrochemicals exposures. Imidacloprid (a), lambda-cyhalothrin (b), pydiflumetofen (c), and tebuconazole (d) exposures resulted in specific alterations to bacterial community.

**Additional file 2.**

## Data Availability

The data that support the findings of this study are available upon reasonable request.

## References

[1] Berg G, Rybakova D, Fischer D, Cernava T, Champomier Vergès M-C, Charles T, Chen X, Coccolin L, Eversole K, Herrero Corral G, Kazou M, Kinkel L, Lange L, Lima N, Loy A, Macklin J A, Maquin E, Mauchline T, McClure R, Mitter B, Ryan M, Sarand I, Smidt H, Schellke B, Roume H, Seghal GK, Selvin J, Correa de Souza R, van Overbeek L, Singh B K, Wagner M, Walsh A, Sessitsch A, Schloter M. Microbiome definition re-visited: old concepts and new challenges. Microbiome, 2020; 8(1): 1-22.

[2] Heong KL, Chen X. Harnessing crop health for the future. Crop Health. 2023;1(1):1. 10.1007/s44297-023-00004-x PMC12825931 41652778

[3] Wang M, Cernava T. Soterobionts: disease-preventing microorganisms and proposed strategies to facilitate their discovery. Curr Opin Microbiol. 2023;75:102349. 37369150 10.1016/j.mib.2023.102349

[4] Singh BK, Trivedi P, Egidi E, Macdonald CA, Delgado-Baquerizo M. Crop microbiome and sustainable agriculture. Nat Rev Microbiol. 2020;18(11):601–2. 33037425 10.1038/s41579-020-00446-y

[5] Xu P, Fan X, Mao Y, Cheng H, Xu A, Lai W, Lv T, Hu Y, Nie Y, Zheng X, Meng Q, Wang Y, Cernava T, Wang M. Temporal metabolite responsiveness of microbiome in the tea plant phyllosphere promotes continuous suppression of fungal pathogens[J]. J Adv Res. 2022;39:49–60. 35777916 10.1016/j.jare.2021.10.003 PMC9263646

[6] Yu P, He X, Baer M, Beirinckx S, Tian T, Moya YAT, Zhang X, Deichmann M, Frey FP, Bresgen V, Li C, Razavi BS, Schaaf G, von Wirén N, Su Z, Bucher M, Tsuda K, Goormachtig S, Chen X, Hochholdinger F. Plant flavones enrich rhizosphere Oxalobacteraceae to improve maize performance under nitrogen deprivation[J]. Nat Plants. 2021;7(4):481–99. 33833418 10.1038/s41477-021-00897-y

[7] Liu X, Matsumoto H, Lv T, Zhan C, Fang H, Pan Q, Xu H, Fan X, Chu T, Chen S, Qiao K, Ma Y, Sun L, Wang Q, Wang M. Phyllosphere microbiome induces host metabolic defence against rice false-smut disease. Nat Microbiol, 2023; 1-15. 10.1038/s41564-023-01379-x 37142774

[8] Berg G, Raaijmakers JM. Saving seed microbiomes. The ISME J. 2018;12(5):1167–70. 29335636 10.1038/s41396-017-0028-2 PMC5931960

[9] Matsumoto H, Fan X, Wang Y, Kusstatscher P, Duan J, Wu S, Chen S, Qiao K, Wang Y, Ma B, Zhu G, Hashidoko Y, Berg G, Cernava T. Bacterial seed endophyte shapes disease resistance in rice. Nat Plants. 2021;7(1):60–72. 33398157 10.1038/s41477-020-00826-5

[10] Nelson EB. The seed microbiome: origins, interactions, and impacts. Plant Soil. 2018;422:7–34.

[11] Shah D, Khan MS, Aziz S, Ali H, Pecoraro L. Molecular and biochemical characterization, antimicrobial activity, stress tolerance, and plant growth-promoting effect of endophytic bacteria isolated from wheat varieties. Microorganisms. 2021;10(1):21. 35056470 10.3390/microorganisms10010021 PMC8777632

[12] Johnston-Monje D, Raizada MN. Conservation and diversity of seed associated endophytes in Zea across boundaries of evolution, ethnography and ecology. PLoS One. 2011;6(6):e20396. 21673982 10.1371/journal.pone.0020396 PMC3108599

[13] Yang L, Danzberger J, Schöler A, Schröder P, Schloter M, Radl V. Dominant groups of potentially active bacteria shared by barley seeds become less abundant in root associated microbiome. Front Plant Sci. 2017;8:1005. 28663753 10.3389/fpls.2017.01005 PMC5471333

[14] Zhan C, Wu M, Fang H, Liu X, Pan J, Fan X, Wang M, Matsumoto H. Characterization of the chemical fungicides-responsive and bacterial pathogen-preventing *Bacillus licheniformis* in rice spikelet[. Food Quality Safety. 2023;7:005.

[15] Zhan C, Matsumoto H, Liu Y, Wang M. Pathways to engineering the phyllosphere microbiome for sustainable crop production. Nat Food. 2022;3(12):997–1004. 37118297 10.1038/s43016-022-00636-2

[16] Liu H, Brettell LE, Singh B. Linking the phyllosphere microbiome to plant health. Trends Plant Sci. 2020;25(9):841–4. 32576433 10.1016/j.tplants.2020.06.003

[17] Wang M, Cernava T. Overhauling the assessment of agrochemical-driven interferences with microbial communities for improved global ecosystem integrity. Environ Sci Ecotechnol. 2020;4:100061. 36157708 10.1016/j.ese.2020.100061 PMC9487991

[18] Lv T, Zhan C, Pan Q, Xu H, Fang H, Wang M, Matsumoto H. Plant pathogenesis: toward multidimensional understanding of the microbiom. iMeta. 2023;2(3):e129. 38867927 10.1002/imt2.129 PMC10989765

[19] Liu X, Fan X, Matsumoto H, Nie Y, Sha Z, Yi K, Pan J, Qian Y, Cao M, Wang Y, Zhu G, Wang M. Biotoxin tropolone contamination associated with nationwide occurrence of pathogen Burkholderia plantarii in agricultural environments in China. Environ Sci Technol. 2018;52(9):5105–14. 29589436 10.1021/acs.est.7b05915

[20] Matsumoto H, Qian Y, Fan X, Chen S, Nie Y, Qiao K, Xiang D, Zhang X, Li M, Guo B, Shen P, Wang Q, Yu Y, Cernava T, Wang M. Reprogramming of phytopathogen transcriptome by a non-bactericidal pesticide residue alleviates its virulence in rice. Fundamental Res. 2022;2(2):198–207. 10.1016/j.fmre.2021.12.012 PMC11197535 38933150

[21] Cao M, Li S, Wang Q, Wei P, Liu Y, Zhu G, Wang M. Track of fate and primary metabolism of trifloxystrobin in rice paddy ecosystem. Sci Total Environ. 2015;518:417–23. 25770954 10.1016/j.scitotenv.2015.03.028

[22] Aloo BN, Mbega ER, Makumba BA, Tumuhairwe JB. Effects of agrochemicals on the beneficial plant rhizobacteria in agricultural systems. Environ Sci Pollution Res; 2021; 1-19. 10.1007/s11356-021-16191-5 34535866

[23] Sun Y, Cao Y, Tong L, Tao F, Wang X, Wu H, Wang M. Exposure to prothioconazole induces developmental toxicity and cardiovascular effects on zebrafish embryo. Chemosphere. 2020;251:126418. 32443233 10.1016/j.chemosphere.2020.126418

[24] Fan X, Matsumoto H, Wang Y, Hu Y, Liu Y, Fang H, Nitkiewicz B, Lau SYL, Wang Q, Fang H, Wang M. Microenvironmental interplay predominated by beneficial Aspergillus abates fungal pathogen incidence in paddy environment. Environ Sci Technol. 2019;53(22):13042–52. 31631659 10.1021/acs.est.9b04616

[25] Li M, Liu T, Yang T, Zhu J, Zhou Y, Wang M, Wang Q. Gut microbiome dysbiosis involves in host non-alcoholic fatty liver disease upon pyrethroid pesticide exposure. Environ Sci Ecotechnol. 2022;11:100185. 36158756 10.1016/j.ese.2022.100185 PMC9488005

[26] da Costa Stuart A K, Stuart RM, Pimentel IC. Effect of agrochemicals on endophytic fungi community associated with crops of organic and conventional soybean (*Glycine max* L. Merril). Agri Nat Res. 2018;52(4):388–92.

[27] Serbent MP, Borges LGA, Quadros A, Marconatto L, Tavares LBB, Giongo A. Prokaryotic and microeukaryotic communities in an experimental rice plantation under long-term use of pesticides. Environ Sci Pollution Res. 2021;28:2328–41. 10.1007/s11356-020-10614-5 32880839

[28] Ramakrishnan B, Venkateswarlu K, Sethunathan N, Megharaj M. Local applications but global implications: Can pesticides drive microorganisms to develop antimicrobial resistance? Sci Total Environ. 2019;654:177–89. 30445319 10.1016/j.scitotenv.2018.11.041

[29] Ruuskanen Suvi, Fuchs Benjamin, Nenonen Riitta, Puigbò Pere, Rainio Miia, Saikkonen Kari, Helander Marjo. Ecosystem consequences of herbicides: the role of microbiome. Trends Ecol Evol. 2023;38(1):35–43. 36243622 10.1016/j.tree.2022.09.009

[30] Hao Z, Wang Y, Guo X, De J. Deciphering the core seed endo-bacteriome of the highland barley in Tibet plateau. Frontiers Plant Sci. 2022;13:1041504. 10.3389/fpls.2022.1041504 PMC9650301 36388601

[31] Lyu Y, Ma S, Liu J, Wang X. A systematic review of highland barley: Ingredients, health functions and applications. Grain Oil Sci Technol. 2022;5(1):35–43.

[32] Wang M, Cernava T. The phyllosphere microbiome. Front Plant Sci. 2023;14:1234843. 37426976 10.3389/fpls.2023.1234843 PMC10325780

[33] Trivedi P, Leach JE, Tringe SG, Sa T, Singh BK. Plant–microbiome interactions: from community assembly to plant health. Nat Rev Microbiol. 2020;18(11):607–21. 32788714 10.1038/s41579-020-0412-1

[34] Vorholt JA. Microbial life in the phyllosphere. Nat Rev Microbiol. 2012;10(12):828–40. 23154261 10.1038/nrmicro2910

[35] Hartmann M, Six J. Soil structure and microbiome functions in agroecosystems. Nat Rev Earth Environ. 2023;4(1):4–18.

[36] Chen X, Wicaksono WA, Berg G, Cernava T. Bacterial communities in the plant phyllosphere harbour distinct responders to a broad-spectrum pesticide. Sci Total Environ. 2021;751:141799. 32889475 10.1016/j.scitotenv.2020.141799

[37] Wan W, Gadd GM, Yang Y, Yuan W, Gu J, Ye L, Liu W. Environmental adaptation is stronger for abundant rather than rare microorganisms in wetland soils from the Qinghai-Tibet Plateau. Mol Ecol. 2021;30(10):2390–403. 33714213 10.1111/mec.15882

[38] Wu W, Logares R, Huang B, Hsieh C. Abundant and rare picoeukaryotic sub-communities present contrasting patterns in the epipelagic waters of marginal seas in the northwestern Pacific Ocean. Environ Microbiol. 2017;19(1):287–300. 27871146 10.1111/1462-2920.13606

[39] Yang Y, Cheng K, Li K, Jin Y, He X. Deciphering the diversity patterns and community assembly of rare and abundant bacterial communities in a wetland system. Sci Total Environ. 2022;838:156334. 35660444 10.1016/j.scitotenv.2022.156334

[40] Soliveres S, Manning P, Prati D, Gossner MM, Alt F, Arndt H, Baumgartner V, Binkenstein J, Birkhofer K, Blaser S, Blüthgen N, Boch S, Böhm S, Börschig C, Buscot F, Diekötter T, Heinze J, Hölzel N, Jung K, Klaus VH, Klein AM, Kleinebecker T, Klemmer S, Krauss J, Lange M, Morris EK, Müller J, Oelmann Y, Overmann J, Pašalić E, Renner SC, Rillig MC, Schaefer HM, Schloter M, Schmitt B, Schöning I, Schrumpf M, Sikorski J, Socher SA, Solly EF, Sonnemann I, Sorkau E, Steckel J, Steffan-Dewenter I, Stempfhuber B, Tschapka M, Türke M, Venter P, Weiner CN, Weisser WW, Werner M, Westphal C, Wilcke W, Wolters V, Wubet T, Wurst S, Fischer M, Allan E. Locally rare species influence grassland ecosystem multifunctionality. Philosophical Transactions Royal Soc B Biol Sci. 2016;371(1694):20150269. 10.1098/rstb.2015.0269 PMC4843691 27114572

[41] Aanderud ZT, Jones SE, Fierer N, Lennon JT. Resuscitation of the rare biosphere contributes to pulses of ecosystem activity. Front Microbiol. 2015;6:24. 25688238 10.3389/fmicb.2015.00024 PMC4311709

[42] Cai H, Wang Y, Xu H, Yan Z, Jia B, Maszenan AM, Jiang H. Niveispirillum cyanobacteriorum sp. nov., a nitrogen-fixing bacterium isolated from cyanobacterial aggregates in a eutrophic lake[J]. International journal of systematic and evolutionary microbiology, 2015; 65(Pt_8): 2537-2541. 10.1099/ijs.0.000299 25944809

[43] Flórez AB, Campedelli I, Delgado S, Alegría Á, Salvetti E, Felis GE, Mayo B, Torriani S. Antibiotic susceptibility profiles of dairy Leuconostoc, analysis of the genetic basis of atypical resistances and transfer of genes in vitro and in a food matrix. PLoS One. 2016;11(1):e0145203. 26726815 10.1371/journal.pone.0145203 PMC4699710

[44] Banerjee S, van der Heijden MGA. Soil microbiomes and one health. Nat Rev Microbiol. 2023;21(1):6–20. 35999468 10.1038/s41579-022-00779-w

[45] Sharma A, Kumar V, Shahzad B, Tanveer M, Sidhu GPS, Handa N, Kohli SK, Yadav P, Bali AS, Parihar RD, Dar OI, Singh K, Jasrotia S, Bakshi P, Ramakrishnan M, Kumar S, Bhardwaj R, Thukral AK. Worldwide pesticide usage and its impacts on ecosystem. SN App Sci. 2019;1:1–16.

[46] Quast C, Pruesse E, Yilmaz P, Gerken J, Schweer T, Yarza P, Peplies J, Glöckner FO. The SILVA ribosomal RNA gene database project: improved data processing and web-based tools. Nucleic Acids Res. 2012;41(D1):D590–6. 23193283 10.1093/nar/gks1219 PMC3531112

