# Supplementary material for: Agrochemical exposure-induced seed microbiome response in barley
Source: Crop Health. 2023 Nov 29;1(1):16. doi: 10.1007/s44297-023-00013-w (PMC12825915; doi:10.1007/s44297-023-00013-w)
Supplement: Supplementary file 1 — Additional file 1: Supplementary Table 1. PerMANOVA test results for internal seed bacterial community in Qingke seeds under different pesticide exposures. Supplementary Fig. 1. Comparative analyses of seed bacteria at genus level in response to various agrochemicals exposures. Imidacloprid (a), lambda-cyhalothrin (b), pydiflumetofen (c), and tebuconazole (d) exposures resulted in specific alterations to bacterial community. [file 44297_2023_13_MOESM1_ESM.docx]

**Supplementary Information**

**Agrochemical exposure-induced seed microbiome response in barley**

Lan Wang^1#^, Hongda Fang^2#^, Zhao Xue^1^, Ji De^1^, Xiaofang Guo^1*^

^1^ School of Environmental Ecology, Tibet University, Lhasa 850000, China

^2^ State Key Laboratory of Rice Biology and Breeding, Zhejiang University, Hangzhou 310058, China

**^*^** To whom correspondence should be addressed:

gxf005@hotmail.com, Tel: 15089021238

^#^ Equally contributed.

**Keyword**

Seed microbiome, agrochemicals, barley, response, ecological risk

**This supplementary information includes:**

**1. Supplementary Table 1**

**2. Supplementary Figures 1**

**Supplementary Table**

Supplementary Table 1. PerMANOVA test results for internal seed bacterial community in Qingke seeds under different pesticide exposures

| Pairs | R^2^ | *P*.value | *P*.adjusted |
| --- | --- | --- | --- |
| CK vs I | 0.07 | 0.041 | 0.195 |
| CK vs L | 0.04 | 0.384 | 0.565 |
| CK vs P | 0.06 | 0.072 | 0.195 |
| CK vs T | 0.06 | 0.051 | 0.195 |

**Supplementary Figures**





**Supplementary Fig. 1 Comparative analyses of seed bacteria at genus level in response to various agrochemicals exposures.** Imidacloprid (a), lambda-cyhalothrin (b), pydiflumetofen (c), and tebuconazole (d) exposures resulted in specific alterations to bacterial community.
